# WT1 Clone 6F-H2 Cytoplasmic Expression Differentiates Astrocytic Tumors from Astrogliosis and Associates with Tumor Grade, Histopathology, IDH1 Status, Apoptotic and Proliferative Indices: A Tissue Microarray Study

**DOI:** 10.31557/APJCP.2020.21.8.2403

**Published:** 2020-08

**Authors:** Amal Abd El Hafez, Heba Salah El Din Ismail Hany

**Affiliations:** *Department of Pathology, Faculty of Medicine, Mansoura University, Mansoura, Egypt. *

**Keywords:** Astrocytic tumors, Astrogliosis, WT1, IDH1, Bcl2, Ki67

## Abstract

**Objectives:**

This tissue microarray (TMA) immunohistochemical (IHC) study elucidates the role of Wilms’ tumor 1 protein (WT1) in diagnosis and prognostication of astrocytic tumors.

**Methods:**

IHC was applied to 75 astrocytic lesions (18 astrogliosis and 57 astrocytic tumors) using antibodies directed against WT1 clone 6F-H2, isocitrate dehydrogenase 1(IDH1), Bcl2 and Ki67. WT1 IHC staining was evaluated and scored blindly by 2 pathologists. Bcl2 and Ki67 scores and labelling indices were assessed and IDH1 status determined. Statistical analysis was performed using the appropriate methodology.

**Results:**

WT1 cytoplasmic expression was detected in 89.5% of astrocytic tumors but not in astrogliosis. Positive WT1 differentiated astrocytic tumors (92.6% accuracy) and grade II diffuse astrocytomas (93.5% accuracy) from astrogliosis with high sensitivity, specificity and positive predictive values (p<0.001). Increased WT1 score significantly associated higher Bcl2 and Ki67 labelling indices, increasing WHO tumor grade and tumor’s histopathologic type (p<0.05) showing staining pattern variability by tumor entity and cell type. Glioblastomas, gliosarcomas and subependymal giant cell astrocytomas were the most frequently WT1 expressing tumors with frequent +3 WT1 score. About 21.4% of pilocytic astrocytomas had +3WT1 score in association with increased Bcl2 and Ki67 indices. Low WT1 scores in grade II and III diffuse astrocytomas were linked to the high frequency of IDH1 positivity, and were associated with low Bcl2 and Ki67 labelling indices. In glioblastomas, WT1 significantly associated poor prognostic variables: older age, negative-IDH1 status, high Bcl2 and Ki67 labelling indices (p=0.04, <0.001, =0.001 and <0.001 respectively).

**Conclusions:**

WT1 clone 6F-H2 is a highly accurate positive surrogate marker to differentiate astrocytic tumors notably the challenging grade II diffuse astrocytoma from astrogliosis. It significantly associates with poor prognostic variables including IDH1 negativity, high apoptotic and proliferative indices and depends on tumor’s histopathologic entity more than its grade. Evaluation of WT1 expression seems essential to tailor patient’s therapy.

## Introduction

A major dilemma in routine practice of surgical neuropathology is to differentiate between reactive astrogliosis and astrocytic tumors particularly low-grade diffuse astrocytomas (Rivera-Zengotita and Yachnis, 2012; Manocha and Jain, 2019). Considering the marked differences in prognosis and therapy, this can be principally challenging with small biopsies that often yield limited amounts of tissue for pathologic study and lack the diagnostic evidences of increased cellularity, mitotic activity, microvascular proliferation or necrosis (Bourne et al., 2010; Rivera-Zengotita and Yachnis, 2012). To serve for this target, new diagnostic methods have been proposed (Kijima et al., 2016; Manocha and Jain, 2019). Of these, is the use of Wilms’ tumor 1 protein (WT1) as a marker to distinguish astrocytic tumors. However, the potential utility of WT1 immunohistochemical (IHC) staining to discriminate reactive astrogliosis from astrocytic tumors and to separate the tumors’ grades is still a subject for controversy (Schittenhelm et al., 2008; Rivera-Zengotita and Yachnis, 2012; Kusum and Ramita; 2019; Manocha and Jain, 2019). It has been suggested that *WT1* gene plays important roles in the tumorigenesis of astrocytic tumors by promoting their malignant phenotype since high-grade tumors usually express higher levels of WT1 proteins (Rushing et al., 2010; Kijima et al., 2016). On the contrary, other studies indicated that WT1 is not a reliable marker to distinguish reactive from neoplastic astrocytes as it is expressed in both (Bourne et al., 2010). 

Being overexpressed in a variety of hematologic malignancies and solid tumors, WT1 has been considered as a molecular target of cancer immunotherapy in several solid tumors and as a tool for monitoring minimal residual disease in leukemic patients (Rushing et al., 2010). Yet, recent clinical trials have investigated WT1 as an immunotherapeutic target and supported its prognostic value and its potential utility as a strong immunotherapeutic target in different types of astrocytic tumors in all age groups even in cases with advanced or recurrent disease (Chiba et al., 2012; Oji et al., 2016; Sakai et al., 2017; Lee et al., 2019; Tsuboi et al., 2019; Sampson et al., 2020). Thus, establishing the immuno-characteristics of WT1 over various astrocytic tumor grades and linking it to the diagnostic and prognostic biomarkers may need further investigation (Bourne et al., 2010; Kijima et al., 2014; Camacho-Urkaray et al., 2018; Manocha and Jain, 2019). Besides, WT1 overexpression needs to be tested before therapy to facilitate clinical decisions. Although the IHC method to assess WT1 mutation appears to be more suitable for routine use on clinical practice, thus far it has not been validated and controversies still exist on its competence (Manocha and Jain, 2019). Therefore, the IHC approach and the potential role of WT1 in astrocytic lesions merit further elucidation.

Using IHC, this study investigates the accuracy of WT1 (clone 6F-H2) to differentiate reactive astrogliosis from astrocytic tumors and to characterize different grades and histopathologic types of astrocytic tumors (according to the WHO classification 2016). Associations between WT1 expression level and the prognostic indicators of astrocytic tumors including: age IDH1 status apoptotic (Bcl2 index) and cell proliferation (Ki67 index) markers are further studied.

## Materials and Methods

This retrospective cohort study was conducted on formalin-fixed, paraffin-embedded (FFPE) tissue blocks obtained from the archives of Pathology Department, Faculty of Medicine, Mansoura University, Egypt. 

*Subjects, inclusion/exclusion criteria*

The study included 75 brain tissue biopsies submitted from the Neurosurgery Department at Mansoura University Hospital (MUH) for pathological diagnosis at Pathology Department, Faculty of Medicine, Mansoura University, Egypt during the period from January 2014 to December 2019. Eighteen specimens diagnosed with reactive astrogliosis and fifty-seven specimens diagnosed with astrocytic tumors of different WHO grades matched the study inclusion criteria: either a stereotactic or excisional biopsy procedure, a histopathological diagnosis of an astrocytic lesion, and availability of sufficient tissue material to apply the study procedure (ideal tissue depth > 2 mm. within block). Astrocytic tumors with significant oligodendroglial component or biopsies with insufficient tissue material were excluded. Patient’s age, gender and biopsy indication/procedure were recorded from the archived pathology request files.

*Histopathologic evaluation*

Routine hematoxylin and eosin (H & E)-stained, 3-4 micrometer-thick, microscopic slides were prepared from all retrieved tissue blocks to be re-evaluated independently by two pathologists to ascertain diagnosis. Cases compatible with the aforementioned inclusion criteria were enrolled. Tumors were classified according to the 2016 WHO classification of central nervous system tumors incorporating the histopathological typing, grading and isocitrate dehydrogenase1 (IDH1) status determination (Louis et al., 2016). 

*Immunohistochemistry (IHC)*

*Tissue microarray (TMA) construction*

TMA blocks will be constructed using Manual Tissue Arrayer (MTA-1, cat.no.MP06, 0.6mm punch-size, Estigen Tissue Science, Estonia). The most appropriate representative area/s in the H&E-stained slides were circled using a pen marker. The corresponding paraffin block-areas were labelled by laying-down the marked H&E slide over the surface of the corresponding block. For each donor block, 4 tissue cores were punched-using a 0.6 mm punch needle- and inserted in 4 consecutive holes in the recipient block following a recorded map. Multiple cores of normal and pathological tissues (kidney, pancreas, lymph node, chorionic villi, lung, colonic mucosa, ovarian serous adenocarcinoma, prostatic hyperplasia, invasive ductal breast carcinoma, colonic adenocarcinoma) were inserted according to the designed map to be set as positive and negative controls for the four-stained IHC antibodies, and to serve as orientation and navigation markers. TMA blocks were heat-incubated in 42°C for 40 minutes put with the sectioning area facing down on a glass slide to seat the tissue cores (Kampf et al., 2012). 

*IHC markers*

About 3-4µm-cut TMA sections (on positively charged silanized VitoGnost SIL adhesive microscope glass slides) were immunostained using the following antibodies:

- Monoclonal mouse anti-human Wilms’ Tumor (WT1): Dako FLEX clone 6F-H2 directed against N-terminal ready to use (Link) code IR055, isotype: IgG1 kappa. Positive control: nuclei of ovarian serous adenocarcinoma and endothelial cell cytoplasm, negative control: lymph node mononuclear cells. Labelled cells display cytoplasmic and/or nuclear staining. 

- Monoclonal mouse anti-human Bcl2 Oncoprotein: Dako FLEX clone124 ready to use (Link) code IR614, isotype: IgG1 kappa. Positive control: mantle zones and T-cell areas of lymph node and invasive ductal breast carcinomas, negative control: nodal germinal centers. Labelled cells display cytoplasmic staining. 

- Monoclonal mouse anti-human Ki67 antigen: Dako FLEX clone MIB1 ready to use (Link) code IR626, isotype: IgG1 kappa. Positive control: colonic mucosal cells and invasive ductal breast carcinomas, negative control: kidney and pancreas. Labelled cells display nuclear staining or chromosomes and cytoplasm in mitotic cells. 

- Rabbit polyclonal anti-human isocitrate dehydrogenase (IDH1): Chongqing Biospes Co. Ltd, cat.no. YPA1706, isotype IgG, Concentrated, 100µg diluted 1:200. Positive control: prostatic hyperplasia, negative control: pancreas. Labeled cells display cytoplasmic staining with granular pattern. 

*IHC procedure*

WT1, Bcl2 and Ki67: IHC was performed with Autostainer Link 48, using its optimized reagents with pharmDx kits EnVisionTM FLEX Visualization Systems (Link code K8000) and EnVision FLEX Hematoxylin (Link code K8008) according to the user’s-guide standardized procedure pre-programmed into the autostainer software. Pre-treatment (dewaxing and dehydration) of FFPE sections with heat-induced epitope retrieval (HIER) using the 3-in-1 specimen preparation procedure was done with these parameters: pre-heat temperature: 65°C; epitope retrieval: 97°C for 20 minutes; cool down to 65°C. The automated protocol is based on an indirect biotin-avidin system and uses a universal biotinylated immunoglobulin secondary antibody and diaminobenzidine (DAB) substrate. After the staining procedure has been completed, the sections were dehydrated, cleared and mounted. 

IDH1: IHC was performed manually using the standard avidin-biotin-peroxidase technique. Tissue sections were deparaffinized and dehydrated through xylene and ethanol, pre-treated with HIER in 0.01 M citrate buffer (pH 6.0) for 10 min. in microwave followed by 3% hydrogen peroxide for 10 min. then incubated with IDH1 antibody for 1 hour at room temperature. DAB was applied for visualization and hematoxylin for counterstaining. 

*Interpretation and scoring of immunostaining*

Immunostaining was visualized by observing sections under light microscope independently by two blinded pathologists, using a semi-quantitative scoring involving both percentage of positive cells and staining intensity. Evaluation considered only the staining of reactive or neoplastic astrocytes excluding the stromal and endothelial cells. WT1 was scored as negative: 0 (0%) or positive: score +1 (<25%), +2 (25-75%), and +3 (>75%) (Mahzouni and Meghdadi, 2012; Kusum and Ramita, 2019), provided that the staining intensity matches at least the intensity of internal control endothelial cells (Schittenhelm et al., 2008). Bcl2 and Ki67 labeling indices were calculated as the percentage of cells with cytoplasmic or nuclear positivity (respectively) divided by the total number of astrocytic nuclei counted, considering the intensity of positive control tissues (Ambroise et al., 2010; Manocha and Jain, 2019). Cutoffs of 10% and 20% were arbitrarily chosen to separate low, intermediate and high labeling indices for Bcl2 (Fels et al., 2000), and Ki67 (Chiba et al., 2010). Strong cytoplasmic IDH1 immunoreaction was considered as positive (Cai et al., 2016). 

*Ethical considerations*

Using FFPE tissue blocks in this retrospective study, we eliminated any influence on biopsy decision or procedure. No further medical interventions were applied to the patients as a part of the study that was conducted upon approval of the committed Institutional Research Board (IRB) at Faculty of Medicine, Mansoura University, Egypt (code number: R.19.12.694). Pathology code numbers of paraffin blocks were used instead of patients’ name to ensure confidentiality and anonymity. All procedures followed the current revision of Helsinki Declaration of medical research involving human subjects (The World Medical Association, 2013). The selected 0.6 mm TMA coring size best preserved the original donor blocks. Finally, the donor blocks were returned to archive for any additional patient’s or investigative use.

*Statistical analysis*

Analysis was performed using IBM Corp. SPSS (International Business Machines Corporation Statistical Product and Service Solutions), released 2013 for Windows, Version 22.0. Armonk, NY: IBM Corp. Continuous variables were presented as mean, median and range (min–max). Categorical variables were expressed as frequencies and percentages. After testing normality using Kolmogorov-Smirnov test. Chi-Square, Fischer exact and Monte Carlo tests were used to compare categorical variables as appropriate. Kruskal Wallis test was used to compare non-parametric continuous variables between WHO grades of astrocytic tumors. Mann-Whitney U test was used to compare 2 independent groups of non-parametric variables. WT1 diagnostic accuracy was detected using cross-tabulation for calculation of sensitivity, specificity, positive and negative predictive values (PPV, NPV) and accuracy after determination of true positive and true negative values. Probability (p-value) <0.05 was considered statistically significant.

## Results

*Demographic and clinicopathological criteria*

The study included 75 astrocytic lesions divided into 2 groups: astrogliosis (18 patients, mean age 40.0±20, range 1-72 years, including 12 males; 66.7% and 6 females; 33.3%) and astrocytic tumors (57 patients, mean age 39.21±19.16, range 4-72 years, including 30 males; 52.6% and 27 females; 47.4%). Both groups were age- and gender-matching (p=0.92 and 0.296 respectively). Concerning astrogliosis, the underlying neuropathology was: adjacent brain tumor; site of previous surgery; chronic brain abscess; cerebral stroke; postnatal hematoma and suspicious malignancy (10; 3; 2; 1;1;1 biopsies respectively).

According to the WHO classification, grade I comprised 31.6% of tumors counting 14 pilocytic astrocytomas (one of which revealed anaplastic features) and 4 subependymal giant cell astrocytomas (SEGAs). Grade II tumors (11;19.3%) included 9 classic diffuse astrocytomas and 2 diffuse astrocytomas with gemistocytic differentiation. Grade III anaplastic astrocytoma comprised 12.3% of tumors (7cases), whereas grade IV was the most frequent (36.8%) encompassing 19 glioblastomas and 2 gliosarcomas. Age, gender, IDH1 status, Bcl2 and Ki67 mean scores and labelling indices revealed significant statistical differences among tumor grades. Low grade (I, II) tumors occurred at younger ages and were more common in females, whereas high grades (III, IV) occurred at older ages and were more common in males (p<0.001 and 0.04 respectively). About 45.6% of tumors attained IDH1 positivity that was demonstrated at higher frequency in grade II and III tumors (90.9 and 85.7% respectively) compared to grade I and IV tumors (11.1% and 38.1% respectively, p=0.001). For grade I, II and III tumors, the mean Bcl2 score ranged from 5 to 8.56% that imposes a low Bcl2 labelling index in most of cases, though 22.2% of grade I tumors had a high index. Grade IV tumors had a mean Bcl2 score of 16.81% and attained mostly intermediate and high indices (38.1% each, p=0.004). All grade I, II and III tumors demonstrated low mean Ki67 scores (1.64 to 4.43%) and mostly had a low Ki67 labelling index in contrast to grade IV that displayed a mean Ki67 score of 16.29% and attained mostly intermediate and high labelling indices (38.1 and 42.9% respectively, p<0.001) (Table 1).

*WT1 accuracy in differentiating all astrocytic tumors and grade II diffuse astrocytoma from astrogliosis*

**Figure 1:**
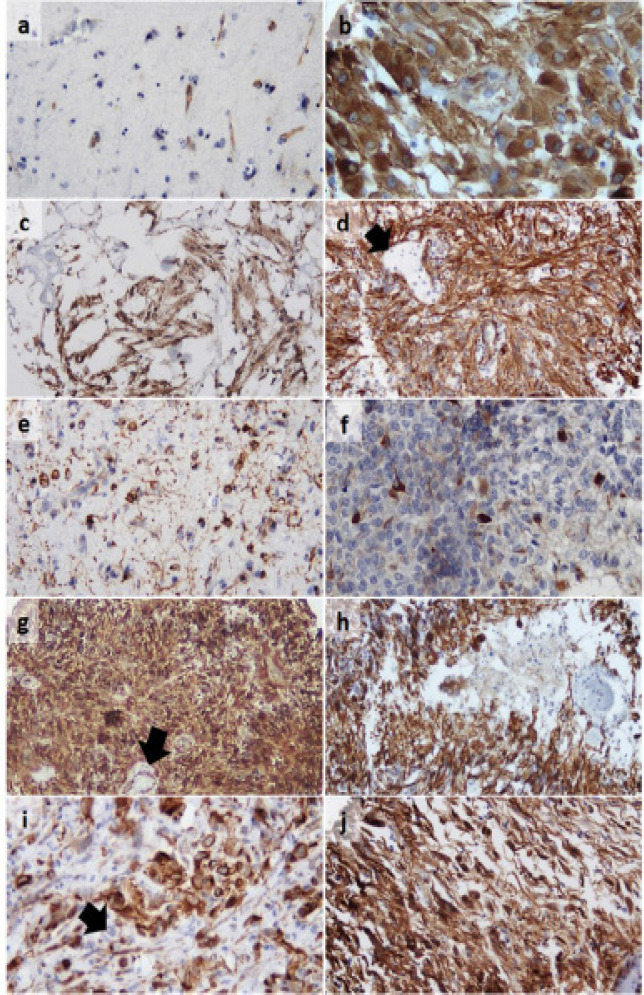
WT1 Expression in Different Astrocytic Lesions. Astrogliosis Showing Negatively-Stained Reactive Astrocytes Compared to Positive Endothelial Internal Control (a, 100x); score +3 subependymal giant cell astrocytoma (b, 200x); pilocytic astrocytomas with score +2 loose plexiform (c, x100) and score +3 dense plexiform patterns (d, 100x); diffuse astrocytoma (e, 100x) and anaplastic astrocytoma showing +1 focal staining pattern (f; 100x); score +3 glioblastoma (g, 40x) showing palisaded positive cells delineating a focus of necrosis (h, 100x); score +2 glioblastoma (i, 100x); and +3 gliosarcoma (j, 100x). WT1 positive luminal endothelial layer but negative proliferating tumor vessels are noted (d, g, i; arrows)

WT1 was expressed by neoplastic astrocytes in 89.5% of astrocytic tumors. Negative tumor samples were 2 pilocytic, 2 grade II diffuse astrocytoma, 1 anaplastic astrocytoma and 1 glioblastoma. None of the reactive astrogliosis samples expressed WT1 in the astrocytic cell population, yet it was only expressed in vascular endothelial cells and some associated immune stromal cells that served as an internal positive control (Figure 1a). There was a highly significant statistical difference in *WT1* expression (p<0.001) with an overall accuracy of 92.6% in differentiating astrocytic tumors from astrogliosis. About 81.8% of grade II diffuse astrocytomas expressed WT1 with a highly significant difference (p<0.001) and diagnostic accuracy of 93.5% when compared to reactive astrogliosis. For both comparisons, WT1 positivity revealed excellent sensitivity (75 and 90% respectively), specificity (100%) and PPV (100%) for astrocytic tumors (Table 2).

*WT1 subcellular localization and staining patterns*

**Figure 2:**
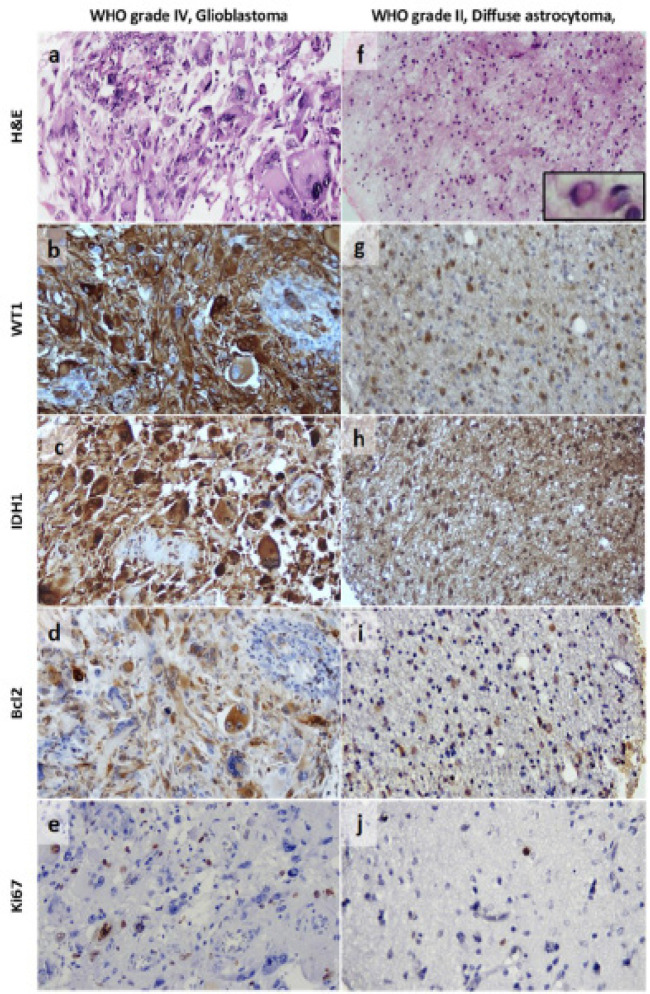
WT1 Score Association with the Prognostic Markers in High- and Low- Astrocytoma Grades. Grade IV, WT1 score+3 glioblastoma with IDH1 positive status, high Bcl2 and high Ki67 indices (a-e, 100x); and a grade II, WT1 score+2 diffuse astrocytoma with gemistocytic differentiation (inset) showing IDH1 positive status, intermediate Bcl2 index (f-i, 100x) and low Ki67 index (j, 200x)

WT1 stained the cytoplasm, astrocytic processes and fibrillary tumor matrix. Infrequently, concomitant nuclear expression was noticed but in a lesser intensity than the cytoplasm. Regarding the staining pattern, a loose or dense plexiform pattern was observed in pilocytic astrocytomas, a focal pattern in grade II diffuse and anaplastic astrocytomas as well as some glioblastomas, while all SEGAs and gliosarcomas and the vast majority of glioblastomas revealed a dense diffuse staining. In glioblastomas, immunoreactivity in palisading tumor cells delineated the negative foci of necrosis. Apart from the WT1-positive luminal endothelial layer, vascular proliferations of glioblastomas and pilocytic astrocytomas were WT1 negative. Certain cell types were strongly and constantly stained including gemistocytic astrocytes, giant cells in SEGA and bizarre or giant cells in glioblastoma (Figures 1 and 2).

*WT1 score distribution in astrocytic tumors and its prognostic associations*

Within 57 astrocytic tumors, 33.3% were evaluated as WT1 score +3, 21.1% as score +2, and 35.1% as score +1, while 10.5% of tumors did not express WT1 (score 0). Amidst different WT1 scores, there was no statistical differences involving the mean patients’ age or gender (p=0.427 and 0.258). The percentages of IDH1 positive and IDH1 negative tumors were almost close per individual WT1 score, yielding a non-significant statistical association (p=0.067). Concerning the Bcl2, a high median score of 20% was detected in WT1 score +3 tumors, in contrast to the lower median scores (6.5, 4.5, 7.5% respectively) detected in 0, +1 and +2 WT1 scores (p<0.001). Therefore, the Bcl2 labelling index significantly associated the WT1 score (p<0.001) as most tumors with low Bcl2 index (59.4%) had a +1WT1 score, while most tumors of intermediate (53.8%) and high (83.4%) Bcl2 indices exhibited a +3WT1 score. For Ki67, a median score of 12% was noticed in WT1 score +3 tumors, in contrast to the overtly lower median Ki67 scores (0, 1, 2% respectively) observed in 0, +1 and +2 WT1 scores (p<0.001). Correspondingly, the Ki67 labelling index was significantly associated with the WT1 score (p=0.034) and most of the low proliferation index tumors (41.7%) lied in the +1 WT1 category, while most tumors of intermediate (41.7%) and high (77.8%) proliferation indices exhibited a +3WT1 score. 

A concordant increase in WT1 score was found with increasing tumor grade (p=0.013). Most grade III and IV tumors were WT1 positive (85.7 and 95.2%). Most grade IV tumors (61.9 and 23.8%) were of WT1+3 and +2 categories. In contrast, most of grade II and III tumors were included in WT1 score+1, followed by score+2 categories. However, 88.9% of grade I tumors were positive of which 33.3% revealed a +3 WT1 score. Likewise, WT1 score was observed to differ significantly according to tumor histopathology (p=0.02). The most frequent immune-positive tumor types were SEGAs and gliosarcomas (100%), followed by the glioblastoma (94.7%). All gliosarcomas, 75% of SEGAs and 57.9% of glioblastomas were WT1 score+3, then 26.3% of glioblastomas and 25% of SEGAs were of WT1 score+2. The least frequently positive histopathologic types were grade II diffuse astrocytoma (81.8%), then pilocytic and anaplastic astrocytomas (85.7% each) and most of these tumor types were of WT1 score+1 (54.5, 50 and 71.4% in the same order). A considerable percentage (21.4%) of pilocytic astrocytomas revealed a WT1+3 staining. 

*Characterization of WT1 expression in astrocytic tumors as distinguished by grade*

**Figure 3:**
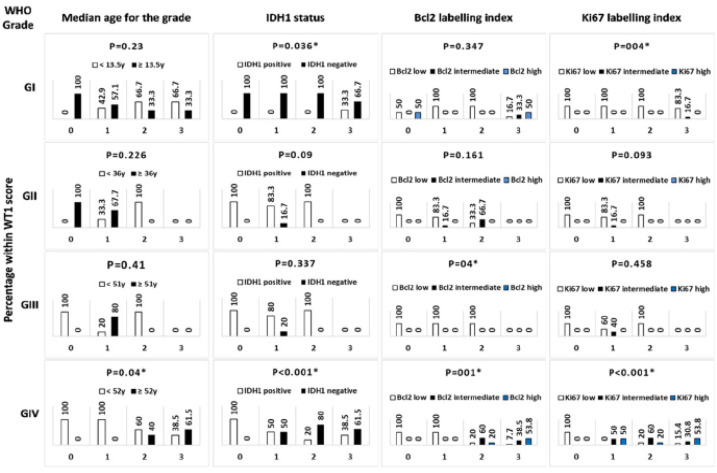
Statistical Analysis of WT1 Expression in Astrocytomas Distinguished by WHO Grade. Bars represent percentages of tumors within each WT1 score (0, +1, +2, +3) displayed in relation to age, IDH1 status, Bcl2 and Ki67 labelling indices respectively. Corresponding p-values are shown, * p-value is significant if <0.05

Figure 3 summarizes the percentages of tumors within each WT1 score and its association with the prognostic variables distinguished by tumor grade. In low-grade astrocytomas (I and II), all WT1 negative cases and most WT1 score +1 were older than the median age for the corresponding grade. In grade I, an association was noticed between IDH1 and WT1 as IDH1 positive tumors (2 tumors) had a WT1+3 score (p=0.036), but this finding was not evident in grade II as the majority of tumors were IDH1 positive and none has a WT1+3 score. WT1+3 tumors of grade I had mostly intermediate or high Bcl2 indices and an intermediate Ki67 index in 16.7% of cases. Meanwhile, all WT1 score 0, +1 and +2 tumors were associated with a low Ki67 index (p=0.4). In grade II, no tumors had high Bcl2 or Ki67 indices and likewise none had WT1 score +3.

In grade III, +1WT1 score involved all the cases who were above the median age or were IDH1 negative. Most grade III tumors were IDH1 positive, and a low and less-likely intermediate Ki67 indices that were distributed with a non-specific paradigm across WT1 scores 0, +1 and +2 rendering insignificant associations. Yet, none of grade III tumors had intermediate or high Bcl2 indices and compatibly, none had WT1 score +3 imparting a significant association (p=0.04). Grade IV astrocytomas demonstrated constantly significant associations between WT1 score and all tested variables. All patients above the median age were included at increasing percentages (40% and 61.5%) in higher WT1 scores +2 and +3 (p=0.04). Similarly, all the IDH-negative tumors were WT1-positive and the percentage of IDH1-negative tumors increased concomitantly within the higher WT1 scores (p<0.001). The percentages of tumors with high and intermediate Bcl2 and Ki67 indices increased with increasing WT1 scores rendering highly significant associations (p=0.001 and p<0.001).

**Table 1:** Clinicopathological Criteria of the 57 Studied Astrocytic Tumors Distributed by WHO Grade

Clinicopathological Criteria	Grade I	Grade II	Grade III	Grade IV	Significance test
	No, (%)	No, (%)	No, (%)	No, (%)	
Histopathology	Pilocytic astrocytoma, 14 (77.8) SEGA, 4 (22.2)	Diffuse astrocytoma	Anaplastic astrocytoma	Glioblastoma, 19 (90.5) Gliosarcoma, 2 (9.5)	
Age/year					
Mean ± SD	17.72±12.755	39.45±10.737	48.14±12.321	54.52±9.822	KW
Median (min–max)	13.5 (4-42)	36 (27-61)	51 (27-67)	52 (39-72)	p<0.001*
Gender (No, %)					
Male (30, 52.6)	5 (27.8)	7 (63.6)	3 (42.9)	15 (71.4)	MC
Female (27, 47.4)	13 (72.2)	4 (36.4)	4 (57.1)	6 (28.6)	p=0.04*
IDH1 status (No, %)					
Positive (25, 45.6)	2 (11.1)	10 (90.9)	6 (85.7)	8 (38.1)	MC
Negative (31, 54.4)	16 (88.9)	1 (9.1)	1 (14.3)	13 (61.9)	p=0.001*
Bcl2 score					
Mean	8.56	5.64	5	16.81	KW
Median (min–max)	5 (0-30)	8 (0-13)	5 (3-8)	12 (0-65)	p=0.02*
Bcl2 index (No, %)					
Low (32, 56.1)	12 (66.7)	8 (72.7)	7 (100.0)	5 (23.8)	MC
Intermediate (13, 22.8)	2 (11.1)	3 (27.3)	0 (0.0)	8 (38.1)	p=0.004*
High (12, 21.1)	4 (22.2)	0 (0.0)	0 (0.0)	8 (38.1)	
Ki67 score					
Mean	2.39	1.64	4.43	16.29	KW
Median (min–max)	1 (0-15)	1 (0-10)	3 (0-12)	15 (0-35)	p<0.001*
Ki67 index (No, %)					
Low (36, 63.2)	17 (94.4)	10 (90.9)	5 (71.4)	4 (19.0)	MC
Intermediate (12, 21.1)	1 (5.6)	1 (9.1)	2 (28.6)	8 (38.1)	p<0.001*
High (9, 15.8)	0 (0.0)	0 (0.0)	0 (0.0)	9 (42.9)	
Total	18 (31.6)	11 (19.3)	7 (12.3)	21 (36.8)	

SEGA, Subependymal giant cell astrocytoma; SD, standard deviation; KW, Kruskal–Wallis test; MC, Monte Carlo test; No, number; * p-value is significant if <0.05

**Table 2:** Accuracy of WT1 Expression in Differentiating Astrocytic Tumors and Grade II Diffuse Astrocytoma in Particular from Reactive Astrogliosis

Astrocytic lesion	WT1 expression (No, %)		Significance test	Sensitivity (%)	Specificity (%)	PPV (%)	NPV (%)	Accuracy (%)
	Positive	Negative						
Astrogliosis	0 (0)	18 (100)						
Astrocytic tumors	51 (89.5)	6 (10.5)	X^2^50.33	89.5	100	100	75	92.6
			p<0.001*					
Grade II diffuse astrocytomas	9 (81.8)	2 (18.2)	MC	81.8	100	100	90	93.5
			p<0.001*					

PPV, positive predictive value; NPV, negative predictive value; No, number, X^2^; Chi-square test; MC, Monte Carlo test; * p-value is significant if <0.05

**Table 3:** Distribution of *WT1* Expression Scores in Astrocytic Tumors and Its Association with the Prognostic Clinicopathological Criteria

Clinicopathological criteria	WT1 expression					Significance test
	No (%)					
	Negative		Positive			
		1	2	3	Total positive	
	6 (10.5)	20 (35.1)	12 (21.1)	19 (33.3)	51 (89.5)	
Age/year						
Mean±SD	35.17±11.48	38.35±18	35.33±18.71	43.84±22.59	39.69±19.89	KW
Median (min–max)	38 (15-47)	42 (8-67)	37 (6-66)	50 (4-72)	42 (4-72)	p=0.427
Gender (No, %)						
Male (30, 52.6)	1 (3.3)	11 (36.7)	6 (20)	12 (40)	29 (96.7)	X^2^=4.04
Female (27, 47.4)	5 (18.5)	9 (33.3)	6 (22.2)	7 (25.9)	22 (81.5)	p=0.258
IDH1 status (No, %)						
Positive (26, 45.6)	4 (15.4)	10 (38.5)	5 (19.2)	7 (26.9)	22 (84.6)	MC
Negative (31, 54.4)	2 (6.5)	10 (32.3)	7 (22.6)	12 (38.7)	29 (93.5)	p=0.067
Bcl2 score						KW
Median (min–max)	6.5 (0-20)	4.5 (0-10)	7.5 (0-20)	20 (0-65)	8 (0-65)	p<0.001*
Bcl2 index (No, %)						
Low (32, 56.1)	5 (15.6)	19 (59.4)	6 (18.8)	2 (6.3)	27 (84.4)	MC
Intermediate (13, 22.8)	0 (0)	1 (7.7)	5 (38.5)	7 (53.8)	13 (100)	p<0.001*
High (12, 21.1)	1 (8.3)	0 (0)	1 (8.3)	10 (83.4)	11 (91.7)	
Ki67 score						KW
Median (min–max)	0 (0-5)	1 (0-20)	2 (0-35)	12 (1-30)	5 (0-35)	p<0.001*
Ki67 index (No, %)						
Low (36, 63.2)	6 (16.7)	15 (41.7)	8 (22.2)	7 (19.4)	30 (83.3)	MC
Intermediate (12, 21.1)	0 (0)	4 (33.3)	3 (25)	5 (41.7)	12 (100)	p=0.034*
High (9, 15.8)	0 (0)	1 (11.1)	1 (11.1)	7 (77.8)	9 (100)	
WHO grade (No, %)						
GI (18, 31.6)	2 (11.1)	7 (38.9)	3 (16.7)	6 (33.3)	16 (88.9)	MC
GII (11, 19.3)	2 (18.2)	6 (54.5)	3 (27.3)	0 (0)	9 (81.8)	p=0.013*
GIII (7, 12.3)	1 (14.3)	5 (71.4)	1 (14.3)	0 (0)	6 (85.7)	
GIV (21, 36.8)	1 (4.8)	2 (9.5)	5 (23.8)	13 (61.9)	20 (95.2)	
Histopathologic type (No, %)						
Pilocytic astrocytoma (14, 24.6)	2 (14.3)	7 (50)	2 (14.3)	3 (21.4)	12 (85.7)	MC
SEGA (4, 7)	0 (0)	0 (0)	1 (25)	3 (75)	4 (100)	p=0.02*
Diffuse astrocytoma (11, 19.3)	2 (18.2)	6 (54.5)	3 (27.3)	0 (0)	9 (81.8)	
Anaplastic astrocytoma (7, 12.3)	1 (14.3)	5 (71.4)	1 (14.3)	0 (0)	6 (85.7)	
Glioblastoma (19, 33.3)	1 (5.3)	2 (10.5)	5 (26.3)	11 (57.9)	18 (94.7)	
Gliosarcoma (2, 3.5)	0 (0)	0 (0)	0 (0)	2 (100)	2 (100)	

SD, standard deviation; No, number, X^2^, Chi-square test; KW, Kruskal–Wallis test; MC, Monte Carlo test; G, grade; SEGA, Subependymal giant cell astrocytoma; *, p-value is significant if <0.05

## Discussion

This study elucidates the role of WT1 as a diagnostic and prognostic marker in neuropathology, particularly for astrocytic tumors. WT1 is zinc finger transcription factor defined as a tumor suppressor gene but also have an oncogenic role that culminates diverse effects on cellular growth, differentiation, migration, invasion, proliferation and apoptosis and confers angiogenic, metastatic and drug resistance abilities to human cancers (Bourne et al., 2010; Clark et al., 2010; Qi et al., 2015; Kijima et al., 2016; Ramsawhook et al., 2018; Salvatorelli et al., 2020). 

A troublesome issue in diagnostic neuropathology is to distinguish astrocytic tumors from astrogliosis, principally in small biopsies involving low-grade infiltrating astrocytomas or infiltration edge of a high-grade tumor. Noticeably, reactive astrocytes undergo hypertrophy and pleomorphism mimicking neoplastic ones (Shao et al., 2016). In this context, several IHC markers were employed such as GFAP, Ki67, p53 and IDH1, but none offered a reliable distinction. GFAP is upregulated in reactive astrocytes, thus it is an excellent IHC marker to differentiate reactive from normal astrocytes, but not from neoplastic ones (Schittenhelm et al., 2008; Pekny et al., 2016; Shao et al., 2016). Moreover, it became evident that p53 is also expressed in a subset of reactive astrocytes and that a significant number of astrocytic tumors lack *p53 *expression. Likewise, not all *TP53* gene mutations result in immunohistochemically detectable p53 (Schittenhelm et al., 2008; Ambroise et al., 2010; Bourne et al., 2010; Camelo-Piragua et al., 2010; Shao et al., 2016). Similarly, Ki67 indices overlap between reactive and neoplastic biopsies of low-grade astrocytomas (Bourne et al., 2010; Louis et al., 2016). Recently, IHC for R132H-mutant IDH1 was proposed to distinguish neoplastic from reactive astrocytes. Nonetheless, IDH mutations in low-grade diffuse astrocytomas ranges from 57 to 88% and almost all pilocytic astrocytomas and primary glioblastomas stains IDH-negative (Camelo-Piragua et al., 2010; Cai et al., 2016; Louis et al., 2016). In this study, less than a half of astrocytic tumors attained IDH1 positivity and the mean Ki67 score in astrogliosis samples was 2.8 (0-15) with 61.1% showing Ki67 positive cells and 11.1% having an intermediate Ki67 index rendering both markers of limited differential value.

In our samples, WT1 was expressed in 89.5% of astrocytic tumors but not in astrogliosis, conferring a diagnostic accuracy of 92.6% for astrocytic tumors and a 100% PPV. Likewise, WT1 differentiated grade II diffuse astrocytoma from astrogliosis with an accuracy of 93.5% and a 100% PPV. However, in WT1-negative lesions the differentiation remained difficult (NPV of 75% and 90%). A previous study established a cut-off value of 0% to recognize neoplastic astrocytes with 100% specificity. The investigators employed WT1 in their diagnostic panel for glial neoplasms and astrogliosis and didn’t report a single false positive result (Schittenhelm et al., 2008). However, some discordance exists in WT1 specificity among earlier studies with a positivity range between 80.9% and 100% in astrocytic tumors (Hashiba et al., 2007; Mahzouni and Meghdadi, 2012; Bassam et al., 2014; Rauscher et al., 2014; Kusum and Ramita, 2019; Manocha and Jain, 2019). In agreement with previous studies (Schittenhelm et al., 2008; Mahzouni and Meghdadi, 2012; Rauscher et al., 2014), we came across 6/57 WT1-negative tumors of different histopathologies, but others didn’t report any negativity in astrocytic tumors (Manocha and Jain, 2019). In unity with the former studies, we can confirm that WT1 expression is limited to neoplastic astrocytes and it can be useful in differentiating astrocytic tumors from astrogliosis. Due to different tissue fixation and IHC methodology, Bourne et al., (2010) detected WT1 in 82% of reactive biopsies and considered it as an unreliable marker for this target. 

Using IHC antibodies against C-terminal, *WT1 *expression was thought to be exclusively nuclear. Upon the development of antibodies against N-terminal (clone WT6F-H2), it became logical to detect WT1 in the cytoplasm, or concurrently in the nucleus and cytoplasm (Salvatorelli et al., 2020). Among our samples, WT1 was detected in the cytoplasm, astrocytic processes and fibrillary tumor matrix with infrequently, concomitant nuclear expression. This cellular localization had been described in preceding reports (Hashiba et al., 2007; Schittenhelm et al., 2008; Bassam et al., 2014; Rauscher et al., 2014; Kusum and Ramita; 2019; Lee et al., 2019). In fact, WT1 is a nuclear/cytoplasmic shuttling protein that transports with polysomes, and a substantial part of WT1 protein localizes in the cytoplasm (Salvatorelli et al., 2020). Our finding, conform with the datum that WT1 is involved in RNA metabolism beside its role as a transcription factor, additionally, overexpression of WT1 isoforms lacking a nuclear localization signal was proposed (Bassam et al., 2014; Lee et al., 2019).

In vitro and in vivo studies have confirmed the oncogenic role of WT1 in human gliomas including astrocytomas (Rauscher et al., 2014). In glioblastoma, WT1 functions as an oncogene by maintaining high proliferative rate and inhibiting apoptosis (Clark et al., 2010; Chen et al., 2011; Kijima et al., 2014). Studies on glioma cell lines have shown that *WT1* silencing with anti-WT1-shRNA (short hairpin RNA) induces apoptosis through upregulation of apoptosis genes as *p53* and PIK3CA (Clark et al., 2010; Kijima et al., 2014; Kijima et al., 2016; Ramsawhook et al., 2018). Furthermore, WT1 functions as a survival and undifferentiation factor in glioblastoma, as *WT1* gene silencing decreases the viability and chemoresistance of glioblastoma cells in vitro (Clark et al., 2010; Chen et al., 2011). Besides, WT1 is suggested to be a potential marker to predict the risk of relapse, progression and patient survival (Qi et al., 2015). 

Recently, there have been trials to associate WT1 expression with several prognostic indicators in human gliomas. Nonetheless, most studies focused on glioblastoma (Kijima et al., 2014; Rauscher et al., 2014; Oji et al., 2016; Camacho-Urkaray et al., 2018). This study analyzed comprehensively WT1 expression across astrocytic tumors. Among 57 tumors, 33.3% were evaluated as WT1 +3, 21.1% as +2, and 35.1% as +1, while 10.5% of tumors did not exhibit WT1 expression. In their study of 87 gliomas, Schittenhelm et al. (2009), described a parallel WT1 score distribution revealing a close correlation between WT1* IHC* expression and WT1 protein in Western blots. In relation to the clinicopathological parameters, we didn’t find any statistical differences across WT1 scores when compared to age, gender or IDH1, although the IDH1-negative tumors tended to have higher frequency and score of WT1 positivity. Therefore, these parameters were further emphasized in separate tumor grades. 

Evading apoptosis is an important step in gliomagenesis. Bcl2 is a 26 kDa-antiapoptotic protein that functions to regulate the outer mitochondrial membrane permeability by blocking proapoptotic proteins like BAX and BAK, thus, it inhibits the mitochondrial release of cytochrome C and preventing apoptosis. In collaboration with WT1/Early Growth Response 1 (EGR1), Bcl2 contributes to dysregulation of calcium homeostasis and signaling in cancer cells. This mechanism seems to control survival and growth of glioblastoma cells (Ambroise et al., 2010; Ritchie et al., 2011; Valdés-Rives et al., 2017). This study disclosed a significant association between increasing WT1 score and the higher Bcl2 scores and labelling indices in astrocytic tumors (p<0.001). Indeed, Bcl2 protein has been reported to be expressed at high levels in malignant gliomas mainly glioblastomas and recurrent gliomas and was found to enhance migration and invasion capability of human glioma cells. In the same vein, Bcl-2 protein overexpression was considered as a negative prognostic factor in anaplastic astrocytoma and as a powerful anti-apoptotic agent in human glioma cultured cells (Fels et al., 2000; Valdés-Rives et al., 2017). 

Ki67-based assessment of proliferative activity is an established method to evaluate tumor’s biology and prognosis. In this regard, we noticed a concordant increase in WT1 score in relation to cell proliferation in astrocytic tumors. Ki67 score and labelling index were significantly associated with the WT1 score (p<0.001 and=0.034 respectively) and most tumors of intermediate and high proliferation indices exhibited a +3WT1 score. A recent study by Manocha and Jain (2019), reported an analogous significant correlation. Likewise, Kusum and Ramita (2019), confirmed the significant direct correlation between mitotic index and WT1 score in astrocytic tumors. The reverse also seems true, as IHC analysis of FFPE tumor sections from astrocytoma animal models transduced with anti-WT1 shRNA revealed that the Ki67 proliferation index was higher in the control tumors (Kijima et al., 2016). Consistent with these data, the role of WT1 as a driver of cell proliferation in astrocytic tumors can be advocated. Albeit, this correlation was attributed to higher WT1 protein expression in areas of high cell proliferation, we agree with Mahzouni and Meghdadi (2012), that higher WT1 protein expression is not necessarily confined to the high proliferation areas. 

The positive correlation between WT1 score and Ki67 labelling index implies its relation to tumor grade (Mahzouni and Meghdadi, 2012). WT1 score increased proportionately with tumor grade (p=0.013). Most grade III and IV tumors were WT1-positive (85.7 and 95.2%) and 61.9% of grade IV tumors had a +3WT1 score. This was in league with other studies reporting a significant increase in mean WT1 score across astrocytoma grades (Hashiba et al., 2007; Mahzouni and Meghdadi, 2012; Bassam et al., 2014; Oji et al., 2016; Camacho-Urkaray et al., 2018; Kusum and Ramita, 2019; Manocha and Jain, 2019), with a frequent assignment of higher WT1 scores to grade IV tumors. Therefore, Manocha and Jain (2019), presumed WT1 utility in differentiating high and lowgrade astrocytomas in challenging biopsies. As WT1 increases significantly with degree of malignancy in astrocytomas, it was linked to poor prognosis (Chiba et al., 2010; Rauscher et al., 2014).

To some extent, WT1 association with tumor grade has been reflected on its histopathology rendering a significant difference (p=0.02) as virtually all gliosarcomas and 94.7% of glioblastomas expressed WT1 and most cases displayed +3WT1 score with diffuse and strong staining. While grade II diffuse astrocytoma was the least frequently positive type and most of diffuse and anaplastic astrocytomas exhibited a focal pattern +1 WT1 score. These data have been established in comparable studies (Schittenhelm et al., 2008; Rauscher et al., 2014; Camacho-Urkaray et al., 2018; Manocha and Jain, 2019), indicating that glial tumors especially of high-grade could be candidates for WT1-targeting cancer immunotherapy. In a striking disparity, 21.4% of our pilocytic astrocytomas and 75% of SEGAs revealed a +3 WT1 staining and virtually all SEGAs were WT1 positive. Moreover, gemistocytes, giant cells in SEGA and bizarre or giant cells in glioblastoma displayed constantly a strong positive staining. In spite of being low-grade tumors, an unexpectedly higher WT1 expression (either in frequency or score) was described in pilocytic astrocytomas and SEGA as compared to grade II diffuse astrocytomas in different studies (Hashiba et al., 2007; Schittenhelm et al., 2008; Schittenhelm et al., 2009; Mahzouni and Meghdadi, 2012; Manocha and Jain, 2019). Moreover, the strong *WT1* expression in gemistocytic, anaplastic and large multinucleated tumor cells has been observed in one study (Schittenhelm et al., 2009). It seems that WT1 expression in brain tumors depends on the tumor’s histopathologic entity more than its grade due to diversity of cellular origin or molecular profile. For emphasis, diffuse (grade II/III) astrocytomas, and secondary glioblastoma share a cell of origin that carry IDH mutation (Cai et al., 2016), while IDH-wild glioblastoma may originate from a bipotential precursor cell, a more primordial neural stem cell or even a dedifferentiated transformed astrocyte (Cohen et al., 2013; Louis et al., 2016). Despite the uncertain histogenesis, SEGA was suggested to arise from neuroglial progenitor cell carrying a biallelic inactivation of Tuberous Sclerosis genes. Moreover, gemistocytic astrocytes exhibit a high frequency *TP53* mutation and gemistocytic astrocytomas more likely undergo progression to anaplastic astrocytoma and glioblastoma (Louis et al., 2016). Likely, pilocytic astrocytomas display significant molecular heterogeneity with BRAF duplication. They harbor a proportion of progenitor-like cells with evidences of proliferation and MAPK activation that are absent from higher-grade gliomas (Reitman et al., 2019). Yet, WT1 association with such cellular and molecular profiles merits further elucidation.

For better characterization, WT1 scores for each grade were studied in comparison to the prognostic variables. In grade I, the most important observations were the occurrence of WT1 low scores in older age patients, the association between the rare IDH1 positivity and +3 WT1 score, the association of intermediate or high Bcl2 and Ki67 indices with +3 WT1 score. Unfortunately, we were unable to compare these findings as *WT1* expression data are limited or not available for astrocytic tumors that predominates in children (Kijima et al., 2016). Grade II and III tumors tended to have low WT1 scores, their age distribution pattern and IDH1 status were non-specific relative to WT1 score, furthermore, neither showed high Bcl2 or Ki67 indices nor a +3WT1 score. In Schittenhelm et al.’s study (2009), no significant differences were observed between grade II and III. Furthermore, Manocha and Jain (2019), attributed the lower WT1 scores in grade II astrocytomas to the high frequency of IDH1 positivity.

In grade IV tumors, WT1 score was significantly higher in older age patients, a finding that has been confirmed previously (Rauscher et al., 2014), providing a link between WT1 expression and poor prognosis in glioblastomas and gliosarcomas. We also observed that, all the IDH1-negative tumors were WT1 positive and had high WT1 scores. It is well-known that secondary glioblastomas predominate in younger patients (median 45 y) and are likely IDH-mutant, while primary glioblastomas arise at older age (median 60 y) and are usually IDH-wild (Cohen et al., 2013; Cai et al., 2016). Taken together, we can confirm that WT1 correlates with old age and IDH1 negativity in grade IV astrocytomas indicating a poor outcome and that, absent/low WT1 expression in high-grade astrocytic tumors is associated with younger age and presence of IDH1 mutation (Rauscher et al., 2014), signifying a favorable prognosis for the latter group (Manocha and Jain, 2019). The inverse relation between WT1 and IDH1 can be simply explained on molecular basis, while IDH1 mutation occurs with increased DNA methylation (hypermethylation phenotype), WT1 was conversely found to be involved in the TET/oxi-mCs (ten eleven translocation/ oxidize 5-methylcytosines)-related demethylation and transcriptional repression resulting in methylome reprogramming and ultimately, tumorigenesis (Cohen et al., 2013; Ramsawhook et al., 2018). On the contrary, other investigators observed higher WT1 scores in the IDH-positive glioblastomas that didn’t reach significant statistical associations (Camacho-Urkaray et al., 2018; Manocha and Jain, 2019). Enduring with the aforementioned role of WT1 in regulating cell proliferation and apoptosis, we found highly significant associations between WT1 score and both Bcl2 and Ki67 indices in grade IV astrocytic tumors, reflecting the aggressive nature and poor prognosis of glioblastomas (Ritchie et al., 2011; Camacho-Urkaray et al., 2018; Tsuboi et al., 2019). 

In conclusion, WT1clone 6F-H2 localizes to neoplastic cell cytoplasm in the vast majority of astrocytic tumors but not in reactive astrocytes. In case of its positivity, WT1 can be used a surrogate marker to differentiate astrocytic tumors notably grade II diffuse astrocytoma from astrogliosis with high accuracy, but is of no value in case of negativity. WT1 score significantly associates higher Bcl2 and Ki67 labelling indices, increasing WHO tumor grade and histopathologic type of astrocytic tumors. It is more frequently and diffusely expressed in glioblastomas, gliosarcomas and SEGAs. A percentage of pilocytic astrocytomas has a high WT1 score that associates increased Bcl2 and Ki67 indices. In glioblastomas, WT1 significantly associates the poor prognostic variables including old age, IDH1 negativity, and high Bcl2 and Ki67 labelling indices. Nonetheless, low WT1 scores in grade II and III tumors can be linked to high frequency of IDH1 positivity and low Bcl2 and Ki67 labelling indices. WT1 is an excellent tumor-associated antigen to target for immunotherapy and WT1 vaccine-based clinical trials have proved safety and efficacy (Sakai et al. 2017; Tsuboi et al. 2019; Sampson et al. 2020). Therefore, evaluation of WT1 expression seems essential to tailor patient’s therapy. 
